# The impact of climate change on photovoltaic power generation in Europe

**DOI:** 10.1038/ncomms10014

**Published:** 2015-12-11

**Authors:** Sonia Jerez, Isabelle Tobin, Robert Vautard, Juan Pedro Montávez, Jose María López-Romero, Françoise Thais, Blanka Bartok, Ole Bøssing Christensen, Augustin Colette, Michel Déqué, Grigory Nikulin, Sven Kotlarski, Erik van Meijgaard, Claas Teichmann, Martin Wild

**Affiliations:** 1 Department of Physics, University of Murcia, 30100 Murcia, Spain; 2 Laboratoire des Sciences du Climat et de l'Environnement (LSCE), IPSL, CEA-CNRS-UVSQ, 91191 Gif sur Yvette, France; 3 Institut de Technico-Economie des Systèmes Energétiques (I-Tésé), CEA/DEN/DANS, 91191 Gif sur Yvette, France; 4 Institute for Atmospheric and Climate Science, ETH Zurich, CH-8092 Zurich, Switzerland; 5 Department of Hungarian Geography, Babes-Bolyai University, 400006 Cluj-Napoca, Romania; 6 Danish Meteorological Institute (DMI), 2100 Copenhagen, Denmark; 7 Institut National de l'Environnement Industriel et des Risques (INERIS), 60550 Verneuil en Halatte, France; 8 Météo-France/CNRM, CNRS/GAME, 31057 Toulouse, France; 9 Swedish Meteorological and Hydrological Institute (SMHI), SE-601 76 Norrköping, Sweden; 10 Royal Netherlands Meteorological Institute (KNMI), 3731 GA De Bilt, The Netherlands; 11 Climate Service Center Germany (GERICS), D-20095 Hamburg, Germany

## Abstract

Ambitious climate change mitigation plans call for a significant increase in the use of renewables, which could, however, make the supply system more vulnerable to climate variability and changes. Here we evaluate climate change impacts on solar photovoltaic (PV) power in Europe using the recent EURO-CORDEX ensemble of high-resolution climate projections together with a PV power production model and assuming a well-developed European PV power fleet. Results indicate that the alteration of solar PV supply by the end of this century compared with the estimations made under current climate conditions should be in the range (−14%;+2%), with the largest decreases in Northern countries. Temporal stability of power generation does not appear as strongly affected in future climate scenarios either, even showing a slight positive trend in Southern countries. Therefore, despite small decreases in production expected in some parts of Europe, climate change is unlikely to threaten the European PV sector.

## Abstract (web summary)

Renewables are key for abating climate change, but also potentially vulnerable to it. Here, the authors show that the power supply from a well-developed European fleet of photovoltaic installations may undergo decreases during the 21st century, but with limited changes in amplitude and temporal stability.

## Graphical abstract

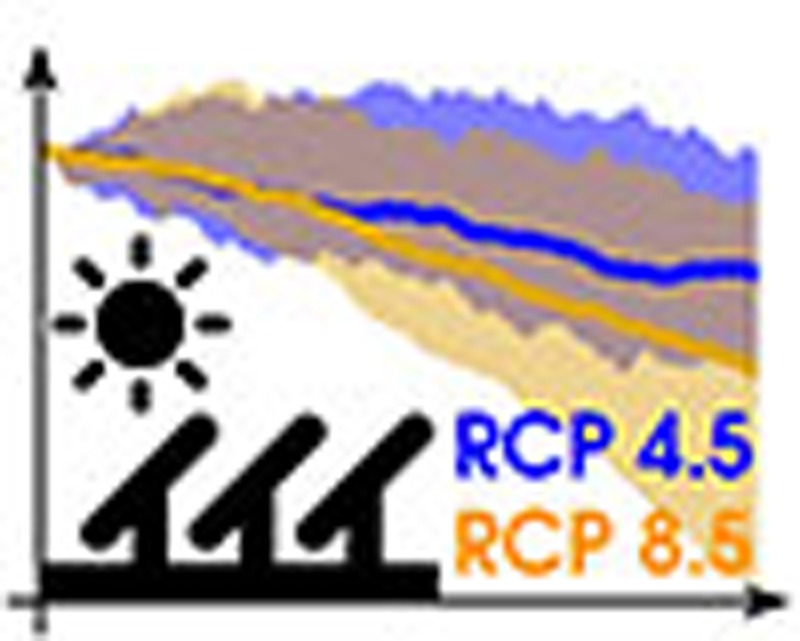

Renewable energies bridge the gap between climate and energy science. This is in part due to the key role played by renewable energies in mitigation strategies aimed at abating climate change and its possible effects on societies and environments^1^. However, most renewable resources are in turn dependent on weather and climate, a dependency (and vulnerability) that could affect the feasibility of future low-carbon energy supply systems.

Photovoltaic (PV) electricity generation depends on solar irradiance, named surface-downwelling shortwave (that is, wavelength interval 0.2–4.0 μm) radiation (RSDS) by climate models, and other atmospheric variables affecting panel efficiency, namely surface air temperature (TAS) and surface wind velocity (VWS). Climate change may therefore affect PV power generation and its temporal stability for a given panel fleet. Estimated changes in potential production (PVpot) based on relatively coarse-resolution global simulations^2^,^3^, including the CMIP5 comprehensive set of global climate projections^4^, indicate small but generally positive impacts of climate change on mean PVpot over Europe either under the A1B SRES scenario^5^ or the RCP8.5 (ref. 6). More local studies found a slight increase in RSDS over the United Kingdom and Greece^7^,^8^ and negligible signals over the North-West of Germany^9^. However, the impact of climate change on PV power generation, including the impact on its temporal stability, considering actual or projected fleets of PV units over an area of the scale of a connected electric grid, such as calculated for wind power^10^, is still lacking.

Our goal here is to provide an overall picture of the direct effects of climate change on solar PV production at the scale of the European regional electric grids considering a future scenario with a strong penetration of PV installations. For that, we use the most up-to-date ensemble of high-resolution regional climate model (RCM) projections: EURO-CORDEX (see Methods and Supplementary Table 1)^11^. In this ensemble, five RCMs were used to downscale five global climate models (GCMs) under two climate scenarios (RCP4.5 and RCP8.5 (ref. 6)). RSDS, TAS and VWS time series retrieved from each ensemble member were used to estimate the corresponding PVpot time series for each grid cell of the domain (see Methods). Next, we first consider changes in PVpot and its drivers (radiation, temperature and wind) for the end of the century, obtaining an asymmetric pattern of slight changes for PVpot, with negative signals northwards and positive southwards, the latter in spite of the non-negligible negative impact of increased temperatures. In a second step, estimates of PV power production considering a PV power fleet with high penetration in Europe are assessed. Results do not show strong impacts on mean production values nor on the temporal stability of the PV power supply under climate change conditions, while overall trends are negative.

## Results

### Changes in PV power generation potential and its drivers

**Figure 1:**
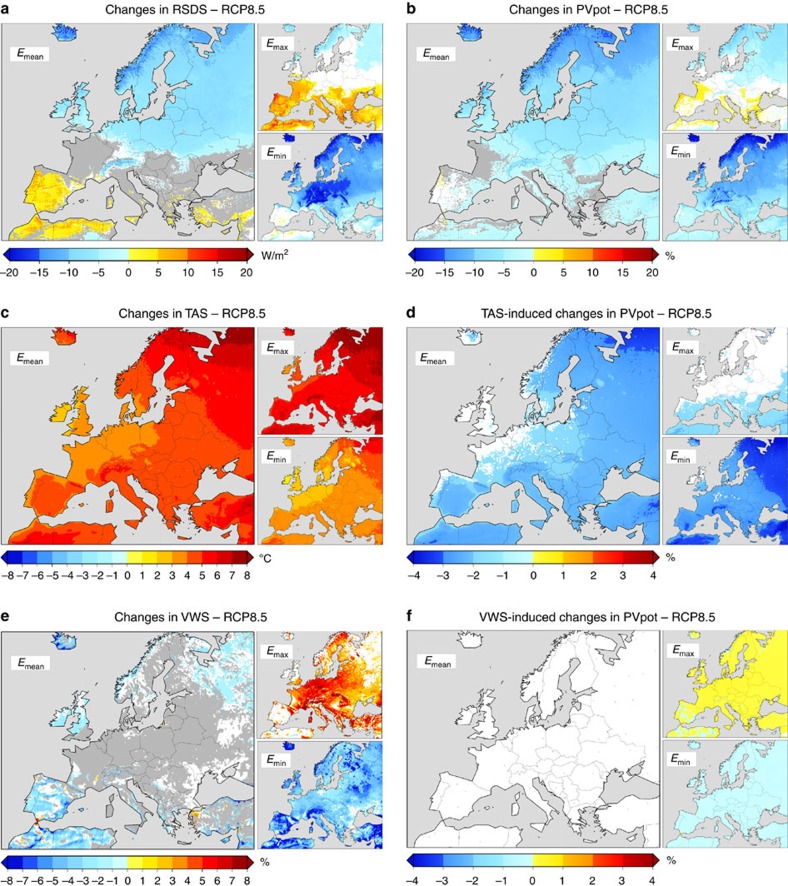
Climate change projections under RCP8.5. Changes projected in the mean values of (**a**) RSDS, (**b**) PVpot, (**c**) TAS and (**e**) VWS under the RCP8.5 to the end of this century (2070–2099 versus 1970–1999) obtained from the *E*_mean_, *E*_max_ and *E*_min_ over land. *E*_max_ and *E*_min_ values are coloured only if they are significant (*p*<0.05) within their corresponding ensemble member, otherwise they are depicted in white. *E*_mean_ values are coloured only if they are robust, in white if they are negligible and in grey if they are uncertain. (**d**,**f**) The *E*_mean_, *E*_max_ and *E*_min_ changes in PVpot that would be induced by the changes in either TAS alone or VWS alone. See the Methods section for details.

The ensemble mean pattern of change for mean RSDS, 2070–2099 versus 1970–1999 climatologies (computed without excluding night-time hours), under the RCP8.5 shows positive signals (about 5 W m^−2^) in Southern Mediterranean regions, negative signals northwards (about −10 W m^−2^, down to −20 W m^−2^ in the northernmost areas) and an intermediate strip where the sign of the change is not robust (Fig. 1a; individual patterns of change in Supplementary Fig. 1). This latitudinal response is typical of that of the North Atlantic Oscillation during its positive phase, which induces windier and cloudier conditions in northern Europe, less windy and cloudy southwards, compared with what prevails during its negative phases^12^,^13^. Thus, the above picture could suggest the imprint of the small but positive trend projected for this large-scale mode of climate variability^14^, since more intense, frequent or persistent positive North Atlantic Oscillation phases would lead to enhanced (depleted) solar radiation in southern (northern) areas within Europe. Weak but significant positive signals expand northwards reaching central regions such as the North of France and Austria, and could occasionally grow up to 20 W m^−2^ in the *E*_max_ (ensemble maximum; see Methods) pattern in Fig. 1a. In the opposite extreme (*E*_min_ pattern in Fig. 1a), negative signals prevail everywhere, with higher amplitude albeit rarely overpassing the limit of −20 W m^−2^.

Similarly, the ensemble mean projected changes for PVpot (Fig. 1b; individual patterns of change in Supplementary Fig. 1) unveil negative values in the entire domain of around −10% under RCP8.5, except for small areas where the mean signal is either negligible (Southwestern Iberia) or of uncertain sign (West of France and coastal areas from Italy to Turkey). The corresponding extreme patterns (*E*_max_ and *E*_min_) show signals up to ±20%. Under RCP4.5, signals are similarly distributed but reduced by about a factor of 2 relative to RCP8.5 (Supplementary Fig. 2). These results frame well with previous findings^2^,^3^,^7^,^8^,^9^, but PVpot changes have a more negative sign in northern and central areas compared with previous works, which can be attributed to the downscaling process since GCMs used in EURO-CORDEX provide a consistently different picture from that of RCMs (Supplementary Fig. 3). In-depth investigation of this feature is beyond the aim of this study, but needs to be addressed in future works to get a higher confidence in the results.

Seasonally (Supplementary Fig. 4), the largest ensemble mean RSDS change signals (expressed in absolute terms, that is, in W m^−2^) occur in spring and summer, when most PV power is produced. The latitudinal pattern (positive southwards and negative northwards) is found in all seasons. These changes, combined with the lower-order effect of changes in TAS and VWS, lead to changes in PVpot that expressed in relative terms (that is, %), are largest in winter (up to −20%) and smallest in summer. However, in summer, the discrepancy in the sign of the change between individual significant signals provides non-robust ensemble mean signals in large areas of Central Europe, a feature not seen in the other seasons.

Regarding the role of TAS and VWS for PVpot projections, it is evident that the positive (negative) changes in radiation are downgraded (strengthened) by the poorer panel efficiency in a TAS-increased scenario (Fig. 1a–d). This is in agreement with previous studies based on different assumptions^2^,^3^,^8^,^15^. While projections for VWS are largely uncertain, having a negligible impact on PVpot (Fig. 1e,f), projections for TAS are positive in the whole domain, varying from 3 to 5 °C in the RCP8.5 ensemble mean pattern, and have associated mean induced changes in PVpot of −3% in the South and East of Europe (see the procedure followed for these estimates in Methods; Supplementary Fig. 2 shows the results for the RCP4.5 case). TAS changes exert the largest influence on PVpot over southern regions in summer, while it is largely negligible in the winter season (Supplementary Fig. 4). Although the projected increase in RSDS largely compensates such TAS-induced effects, these results call for future efforts to reduce the dependence of the PV cells performance on the ambient temperature, as already stated in ref. 16.

### Changes in PV power production per region

**Figure 2:**
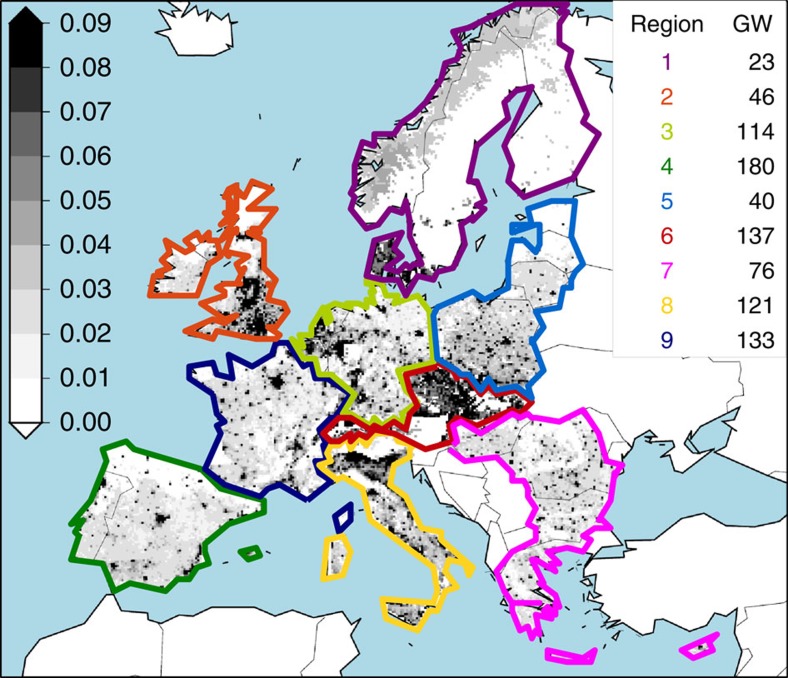
Estimated spatial distribution of the 2050 PV power fleet. In grey shadows: spatial density (in %) of the distribution of the PV power-installed capacity by the year 2050 as obtained with the CLIMIX model over the EURO-CORDEX spatial grid considering the regional targets proposed by the European Climate Foundation in its 80% RES pathway^7^. These targets are provided as GW of installed PV power capacity per region, as listed, with each region comprising either one or several countries, namely: Denmark, Finland, Norway and Sweden compose region 1; Ireland and UK, region 2; Belgium, Germany and The Netherlands, region 3; Portugal and Spain, region 4; Estonia, Latvia, Lithuania and Poland, region 5; Austria, Czech Republic, Slovakia, Slovenia and Switzerland, region 6; Bulgaria, Cyprus, Greece, Hungary and Romania, region 7; Italy and Malta, region 8; and France, region 9.

**Figure 3:**
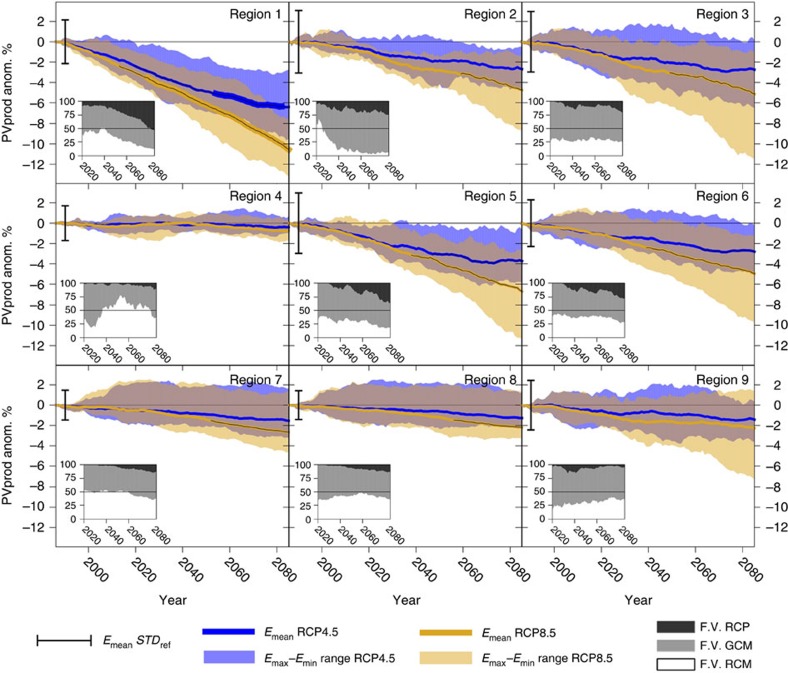
Time series of PV power production along the 21st century under both RCP4.5 and RCP8.5. Thirty-year running mean time series of the estimated PV power production anomalies under both the RCP4.5 (blue) and the RCP8.5 (orange) in each region. Anomalies are computed with respect to the mean values in the reference period 1970–1999 and expressed in %. Solid lines depict the ensemble mean values, with the widest segments, appearing only in the first plot (region 1), indicating S2N>1. Shadows show the ensemble spread. Vertical black bars depict 0±the ensemble mean value of the standard deviation of the annual series of PV power production anomalies in the reference period, as representative of the current natural variability. If the ensemble mean change exceeds such a quantity, a thin black line is superimposed on the ensemble mean series. Small subplots depict the fraction of variance (in %) explained by the change of RCP (dark-grey shadow), GCM driving run (light-grey shadow) or RCM (white shadow), as obtained from an analysis of variance applied to the whole set of 30-year running mean time series of PV power generation anomalies considering only the scenario period. See the Methods section for details.

**Figure 4:**
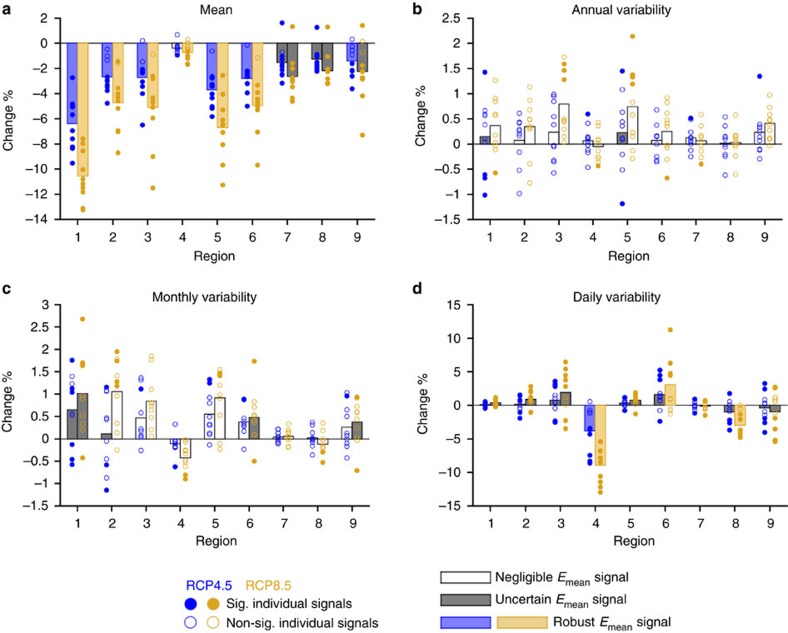
Projected changes in the mean and the time variability of the PV power production series. Changes projected to the end of this century (2070–2099 versus 1970–1999) in (**a**) the mean, (**b**) the annual variability, (**c**) the monthly variability and (**d**) the daily variability of the PV power production series of each region under both the RCP4.5 (blue) and the RCP8.5 (orange). The individual signals corresponding to each ensemble member are depicted by circles: filled if they are significant, empty if not. Boxes represent ensemble mean signals: coloured, white or grey if robust, negligible or uncertain, respectively. See the Methods section for details.

Going a step further to evaluate changes in actual PV power generation, we now make assumptions on where the PV power capacity is/will be installed in Europe. For that, the CLIMIX model^17^ is applied to the regional objectives for the PV sector proposed by the European Climate Foundation Roadmap 2050 (ref. 18) in the 80% renewable energy supply (RES) pathway (Fig. 2; see Methods for more details). Under such a fixed scenario of high-PV penetration, a general and progressive decline of the generated PV power is found in all regions along the entire period (Fig. 3). This is most pronounced in the northernmost region 1, with ensemble mean projected changes up to −6% and −10% under RCP4.5 and RCP8.5, respectively. In Northern, Western and Central Europe (regions 1, 2, 3, 5 and 6) changes of −3 to −6% are obtained by the end of the century. In Southern Europe, changes are smaller (see also Fig. 4a). Note that the fact that changes are expressed here in relative terms partly contributes to obtain the smallest changes over regions and seasons (see also Supplementary Fig. 5) with the highest potential (that is, southwards, summertime).

Except for the regions 4 and 9, the mean projected changes under RCP8.5 exceed the current interannual variability of the PV production series from 2050 onwards (we will call this detectability of the mean change hereafter; see Methods). Under RCP4.5, only the regions 1, 5 and 6 display detectable mean changes from 2050 onwards, and regions 2 and 3 at the very end of the century (Fig. 3; Supplementary Fig. 6). However, in all cases the magnitude of the ensemble mean projected changes, even for those noted as robust in Fig. 4a, is smaller than the ensemble spread, indicating a relatively high degree of uncertainty compared with the magnitude of the ensemble mean signals. Only for region 1, and especially under RCP8.5 towards the end of the century, the signal-to-noise ratio (S2N; see Methods) exceeds 1 (Fig. 3). In regions 2, 3, 5 and 6, with mean changes of about 5% and spreads of 10% by the end of the period, S2N falls down to as far as 0.5 (Supplementary Fig. 6). It drops to even below 0.5 in regions 4, 7, 8 and 9 (Supplementary Fig. 6), although this is neither surprising nor very relevant because mean changes in these regions are (1) close to zero, thus providing a close-to-zero value for the numerator of the S2N ratio, and (2) generally non-robust, that is, the spread mostly involves non-significant signals.

To better understand the aforementioned uncertainty, a two-step one-way analysis of variance^19^ is applied to the set of time series of PV power production anomalies to discern the roles played by the RCP, the GCM and the RCM choice. Results (shown in the small subplots of Fig. 3) unveil that the choice of the RCP exerts a small role in explaining the variance among the whole set of time series compared with the GCM+RCM-induced spread, while its influence grows along the century. Overall, the influence of the GCM prevails in the northernmost regions, while in the southern and central regions the RCM plays a dominant role. These latter regions, namely 4, 6, 7, 8 and 9, are located in South-central Europe, where regional feedbacks involving temperature, soil moisture and clouds are particularly important^20^,^21^,^22^, inducing a higher variety of model responses than in northern areas with weakly constrained land–atmosphere interactions^23^. Besides, these southernmost areas have a highly complex orography, which has a strong impact on the simulated regional climates. Worth to note that, while RCMs thus appear more tightly constrained by their driving GCM northwards than southwards, RCMs are able to turn the sign of the projected change in the resource from positive to negative in some northern areas (Supplementary Fig. 3). However, as it occurs similarly for all RCMs driven by the same GCM, it is not reflected in the results of the analysis of variance.

Finally, we focus on a key aspect of all weather-dependent renewable energies concerning their grid integration and management: the temporal stability of their supply. Our results indicate that changes in the monthly and annual variability of the PV production series (see Methods) are overall non-significant (Fig. 4b,c), while significant reductions of the interannual variability in the winter series of eastern regions appear in the seasonal analysis (Supplementary Fig. 5e). Although in some cases the ensemble spread leads to uncertainty concerning the mean signals, projected changes in variability at these timescales (annual and monthly) of each of the models separately remain in the range (−1.5%;+3%) (Fig. 4b,c). By contrast, robust decreases are found for daily variability in a few regions, namely 4 and 8 (ranging from −3 to −9% to the end of the century depending on the region and the RCP), indicating a slightly higher temporal stability of the daily PV production (Fig. 4d). This is most evident in region 4, where it occurs in all seasons but winter (Supplementary Fig. 5m–p) and despite the fact that the daily variability of TAS is projected to increase there, being therefore lead by the projected reduction of the daily variability of RSDS (Supplementary Fig. 7). In the central region 6, however, a robust positive signal for changes in daily variability is found assuming RCP8.5 (Fig. 4d). These changes would occur progressively along the entire period, being consistently smaller by mid-century (Supplementary Fig. 8).

## Discussion

Climate change is therefore projected to affect PV power production by a reduction of at most 10–12% in Scandinavian areas where, admittedly, solar PV is not expected to become the main source of renewable energy^18^. In southern areas, in contrast, slight increases in the mean PV supply and in its daily stability were found. Uncertainties still remain in our assessment though, due for instance to indirect effects of natural and anthropogenic aerosols^24^ and to land-use changes, both features being currently poorly represented or totally ignored in RCMs, and to the tilt of PV panels, a fact not considered here. Also, whereas the effect of ambient conditions on the PV cell temperature and, thereby, performance has been explicitly accounted for here, other factors that may affect the outdoor performance of PV modules, such as the solar spectrum distribution and the airmass effect on it^25^,^26^, are absent in our analysis. These unrepresented factors may have an impact on the reported signals for northern areas, which will suffer more from the low irradiance performance of PV modules than sites in the south of Europe where the solar irradiance retains higher values and the PV cell temperature effect prevails^27^. Besides, we found somewhat conflicting signals when comparing GCM and RCM-downscaled results regarding PVpot projections, which needs further investigation. Nevertheless, and although it should be acknowledged that climate models tend to underestimate the multidecadal variations in RSDS^28^,^29^, indicating the potential for larger changes than currently projected, none of the simulations analysed so far, here or elsewhere^2^,^3^,^7^,^8^,^9^,^15^,^24^, provides strong changes. Not even, or even less, if we incorporate the assumption of a well-developed PV power supply system across Europe. Actually, the fact that PV systems are foreseen to largely expand over the 21st century, together with other technological and politico-economical aspects (for example, increased lifetime of PV modules, module price decrease and appropriate policies supporting PV system deployment)—all non-physical aspects determining the evolution of the PV energy market^30^,^31^, as discussed in ref. 24—should amply counteract the reported climate change-derived negative signals for the resource availability. Therefore, climate change hardly compromises the European development of PVs.

## Methods

### Climate simulations

Two ensembles of regional climate simulations spanning the period 1970–2099 were used in this study. Both comprise the same 10 members (involving six different RCMs and five GCM runs; Supplementary Table 1), but one assumes the moderate RCP4.5 and the other the more marked RCP8.5 (ref. 6). The simulations were performed under the umbrella of the EURO-CORDEX project^11^,^32^, cover Europe with a spatial resolution of 0.11° both in latitude and longitude, the finest so far in this type of climatological multi-model and multi-scenario experiments, and provide records of the output variables every 3 h. The ensemble mean climatologial patterns of all the magnitudes utilized in this study, that is, mean values and variability of several variables at various timescales, are provided in Supplementary Figs 9 and 10 as a referential baseline.

A concise evaluation exercise of the simulated RSDS, further supported by the results presented in ref. 33, is also included in Supplementary Fig. 9. All ensemble members are well able to capture both the spatial distribution and variability of the observed mean RSDS pattern, which is mainly dominated by a latitudinal gradient both in models and observations. Small negative errors (below 10%) appear in a few points mainly in the Iberian Peninsula, while overestimation errors prevail elsewhere reaching up to 20%. Excessive RSDS is a long-standing problem in climate modelling^34^,^35^. There is, however, a number of points where positive biases grow even beyond 40%, usually located in mountainous regions, such as the Alps, where the limitation of climate models to reproduce single measurements is well known. Regarding RSDS variability, ensemble mean errors at the annual and monthly (daily) timescales are generally negative and mostly below 2% (10%), rarely up to 6% (15%). However, while the spatial distribution of the simulated monthly and daily variability patterns is acceptable, it is not the case for the annual variability simulated pattern. Nonetheless, taking into account (1) the small number of observational points, (2) the very nature of models that perform a spatial discretization of the simulation domain, along with the actual spatial representativeness of ground-based solar radiation measurements^36^, (3) the still moderate accuracy of models to properly reproduce cloudiness^37^ and (4) the lack of a dynamically modelling of aerosols concentration levels, the results of this validation exercise are within an acceptable range, thus allowing a moderately high confidence on the analysed simulations.

### PV power potential and production

The PV power output at a site depends on two factors: its PV power generation potential (PVpot) and the installed capacity. As defined and used in this study, PVpot is a dimensionless magnitude accounting for the performance of the PV cells with respect to their nominal power capacity according to the actual ambient conditions. Therefore, PVpot multiplied by the nominal installed watts of PV power capacity gives instantaneous PV power production.

PVpot mainly involves the amount of the resource (RSDS) but also the influence that other atmospheric variables may have on the efficiency of the PV cells, which diminishes as their temperature increases^38^. According to the literature^39^, it can be expressed as:


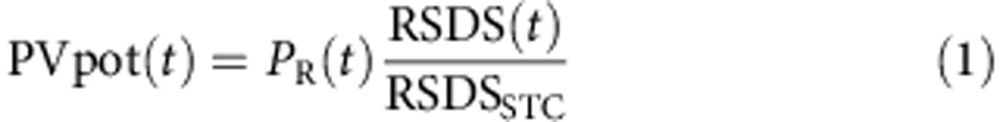


where STC refers to standard test conditions (RSDS_STC_=1,000 W m^−2^), those for which the nominal capacity of a PV device is determined as its measured power output, and *P*_R_ is the so-called performance ratio, formulated to account for changes of the PV cells efficiency due to changes in their temperature as:


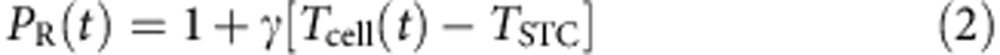


where *T*_cell_ in the PV cell temperature, *T*_STC_=25 °C and *γ* is taken here as −0.005 °C^−1^, considering the typical response of monocrystalline silicon solar panels^40^. Finally, *T*_cell_ is modelled considering the effects of TAS, RSDS and VWS on it as:


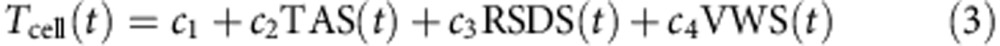


with *c*_1_=4.3 °C, *c*_2_=0.943, *c*_3_=0.028 °C m^2^ W^−1^ and *c*_4_=−1.528 °C sm^−1^ according to ref. 41.

Hence, if ambient conditions (RSDS, TAS and VWS) correspond to the STCs, PVpot equals 1 and PV power production reaches the rated value. If they are so that *T*_cell_ is higher (lower) than 25 °C and/or RSDS lower (higher) than 1,000 W m^−2^, PVpot will be lower (higher) than the unit and the PV power output will be lower (higher) than the nominal power of the module.

The spatial distribution of PV power-installed capacity was obtained with the CLIMIX model as in ref. 17. Here it is applied to the 2050 regional targets set by the European Climate Foundation Roadmap 2050 in its 80% RES pathway^18^ using the 0.11°-resolution working grid defined for EURO-CORDEX (Fig. 2). CLIMIX performs an optimization exercise based on the resource availability for allocating PV plants (that is, by default, the preferred locations are those where RSDS is most abundant), but without overloading the best locations with huge deployments of PV installations, discarding sites previously identified as forestry or inaccessible areas and taking into account the population distribution.

Using the PVpot time series estimated as described above and the 2050 spatial scenario of PV power-installed capacity, 3-h time series of PV power generation were obtained for each grid cell of the domain holding PV power units. These series were finally aggregated per region, considering those regions for which the aforementioned 2050 targets were specified (Fig. 2). As a result, nine 3-h time series of PV power generation spanning the period 1970–2099 were obtained, one per region, from each one of the available simulations. Ensemble mean values of their means and standard deviations (as computed here to assess changes in time variability) are presented in Supplementary Fig. 11 as a reference.

### Analysis of time variability

Time variability is assessed here at various timescales (daily, monthly and annual) as follows. First, the 3-h series of PV power generation are daily, monthly and yearly aggregated and then detrended to avoid capturing long-term changes rather than those occurring at the scales of interest. In addition, multi-year monthly and daily means are removed from the monthly and daily series, respectively, to avoid the masking effect of the annual cycle of PVpot. Finally, annual, monthly and daily variability is just computed as the normalized standard deviation of the resulting series and expressed here in %. The normalization consists on dividing by the climatology of the historical period (that is, the 1970–1999 mean values) and was not performed in the case of the TAS series due to the very nature of this variable. The described procedure was applied either for the whole series or for the seasonally split series.

### Assessment of projected changes

Climate change signals from each individual ensemble member are computed as the difference between the value of the assessed magnitude in a 30-year-long future period (namely, 2070–2099) and its value in the reference period 1970–1999. The statistical significance of changes is evaluated using the Student's *t*-test imposing *p*<0.05. For mean PVpot, mean VWS and mean PV power production, changes are expressed in relative terms (% with respect to the reference climatology); otherwise changes represent just the difference between future and historical values. Note that since variability is expressed here in %, variability changes are also given in %, but they do not represent variations in relative terms.

The ensemble mean (*E*_mean_) signals are computed as the arithmetic mean of the set of individual signals within each ensemble. The following criteria are used to consider them as either uncertain or negligible: at least two significant individual signals differ in the sign of the projected change (uncertain); the uncertainty condition is not fulfilled and less than a half of the individual signals are significant (negligible). Otherwise, the ensemble mean signals are referred to as robust.

In addition, ensemble maximum and minimum (*E*_max_ and *E*_min_, respectively) signals are computed as the maximum and minimum values, respectively, of the whole set of individual signals. The ensemble spread denotes the range between these two limit values.

### TAS- and VWS-induced changes in PV power generation potential

By grouping equations (1)–(3), 2, 3, the expression of PVpot can be rewritten as:


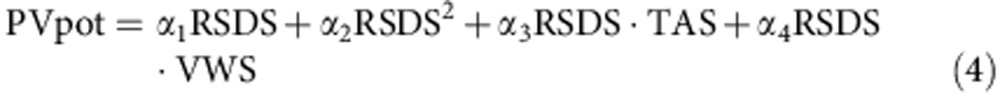


with *α*_1_=1.1035 × 10^−3^, *α*_2_=−1.4 × 10^−7^, *α*_3_=−4.715 × 10^−6^ and *α*_4_=7.64 × 10^−6^ in the corresponding units (PVpot should be dimensionless). Thereby, changes in PVpot are given by:


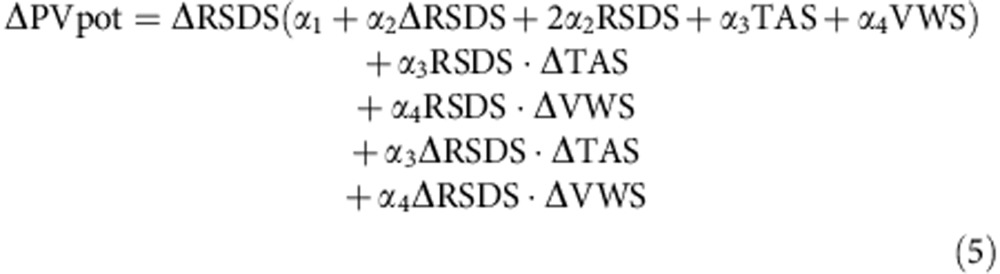


Hence, taking ΔRSDS=ΔVWS=0 in equation (5), the change in PVpot due to a change in TAS can be obtained. Analogously, the change in PVpot due to the influence of changes in VWS alone is given by imposing ΔRSDS=ΔTAS=0 in equation (5). This is the procedure adopted here, as it was done in previous works^2^,^3^,^8^. However, note that the cross-products in the last two terms of equation (5) make it impossible to fully isolate the contribution of each single field.

### Detectability and S2N of projected changes

Two additional measures were employed to elucidate the forcefulness of the ensemble mean projected changes in PV power generation. The signal-to-noise ratio (S2N) refers to the ratio between the magnitude of the ensemble mean change and the ensemble spread. Detectability describes the ratio between the magnitude of the ensemble mean change and the ensemble mean annual variability of the PV power generation series. Therefore, a value of this ratio larger than unity implies that the magnitude of the ensemble mean change falls beyond the expected natural variability of the series.

## Additional information

**How to cite this article:** Jerez, S. *et al.* The impact of climate change on photovoltaic power generation in Europe. *Nat. Commun.* 6:10014 doi: 10.1038/ncomms10014 (2015).

## Supplementary Material

Supplementary Information Supplementary Figures 1-11, Supplementary Table 1 and Supplementary References
