# Supplementary material for: The impact of climate change on photovoltaic power generation in Europe
Source: Nat Commun. 2015 Dec 11;6:10014. doi: 10.1038/ncomms10014 (PMC4682048; doi:10.1038/ncomms10014)
Supplement: Supplementary Information — Supplementary Figures 1-11, Supplementary Table 1 and Supplementary References [file ncomms10014-s1.pdf]

Supplementary Figure 1. Individual climate change projections under RCP8.5.

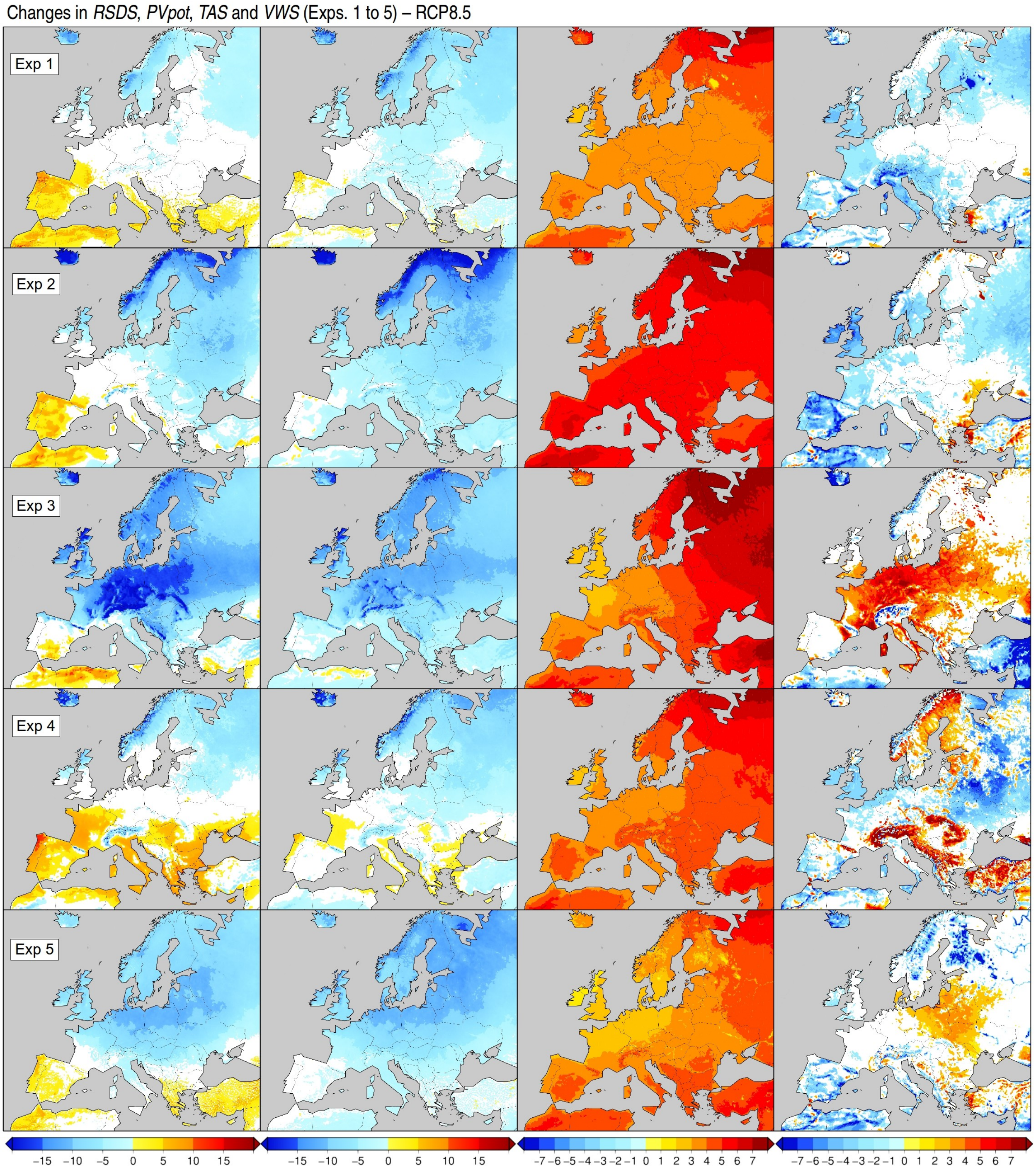

Changes projected in the mean values of *RSDS* (1st column, in Wm^{-2}), *PVpot* (2nd column, in %), *TAS* (3rd column, in ºC) and *VWS* (4th column, in %) under the RCP8.5 to the end of this century (2070-2099 *vs*. 1970-1999) by each ensemble member. Non-significant signals (p>0.05) are depicted in white. (Continued on next page)

Supplementary Figure 1. (Continuation)

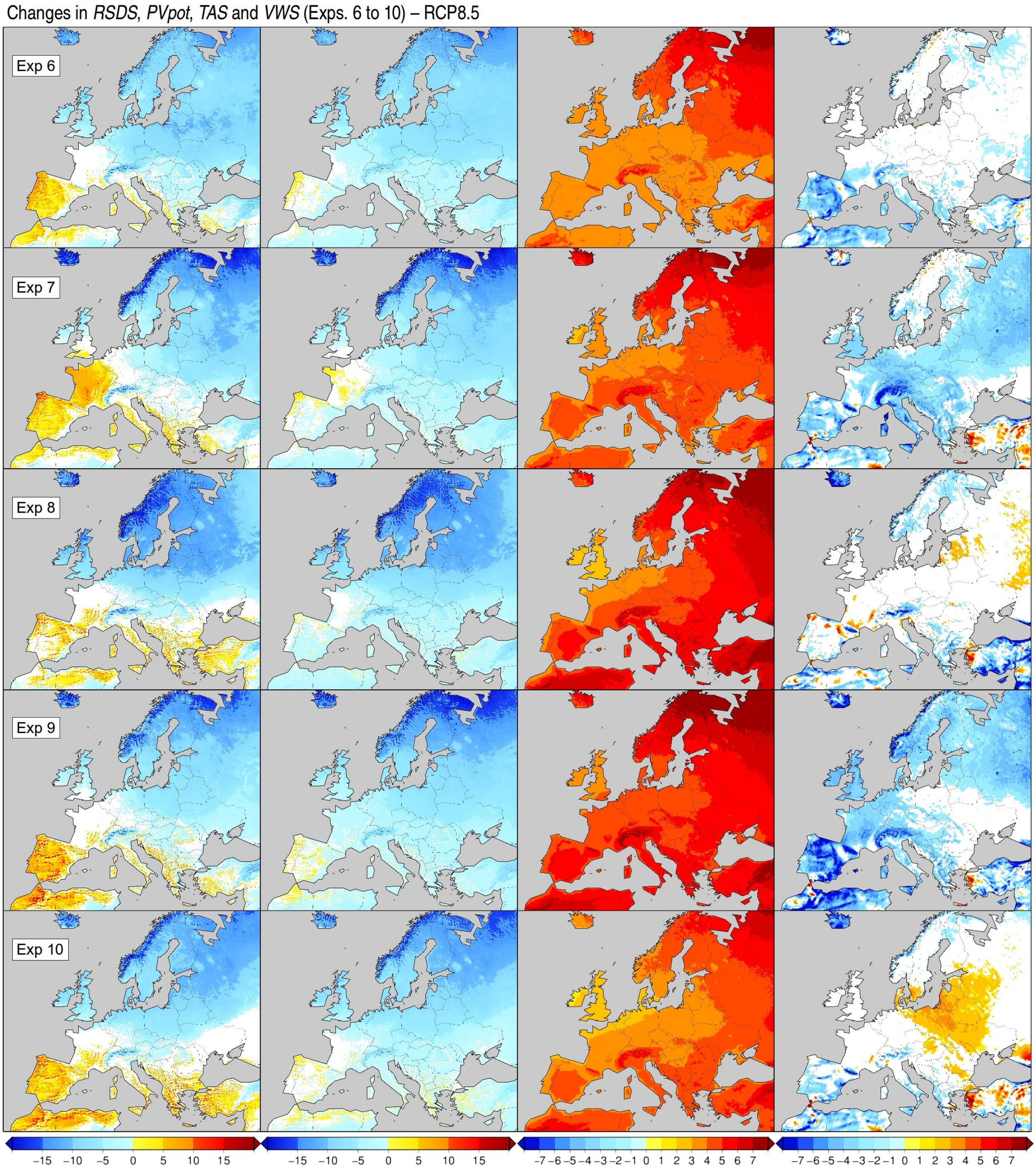

Supplementary Figure 2. Climate change projections under RCP4.5.

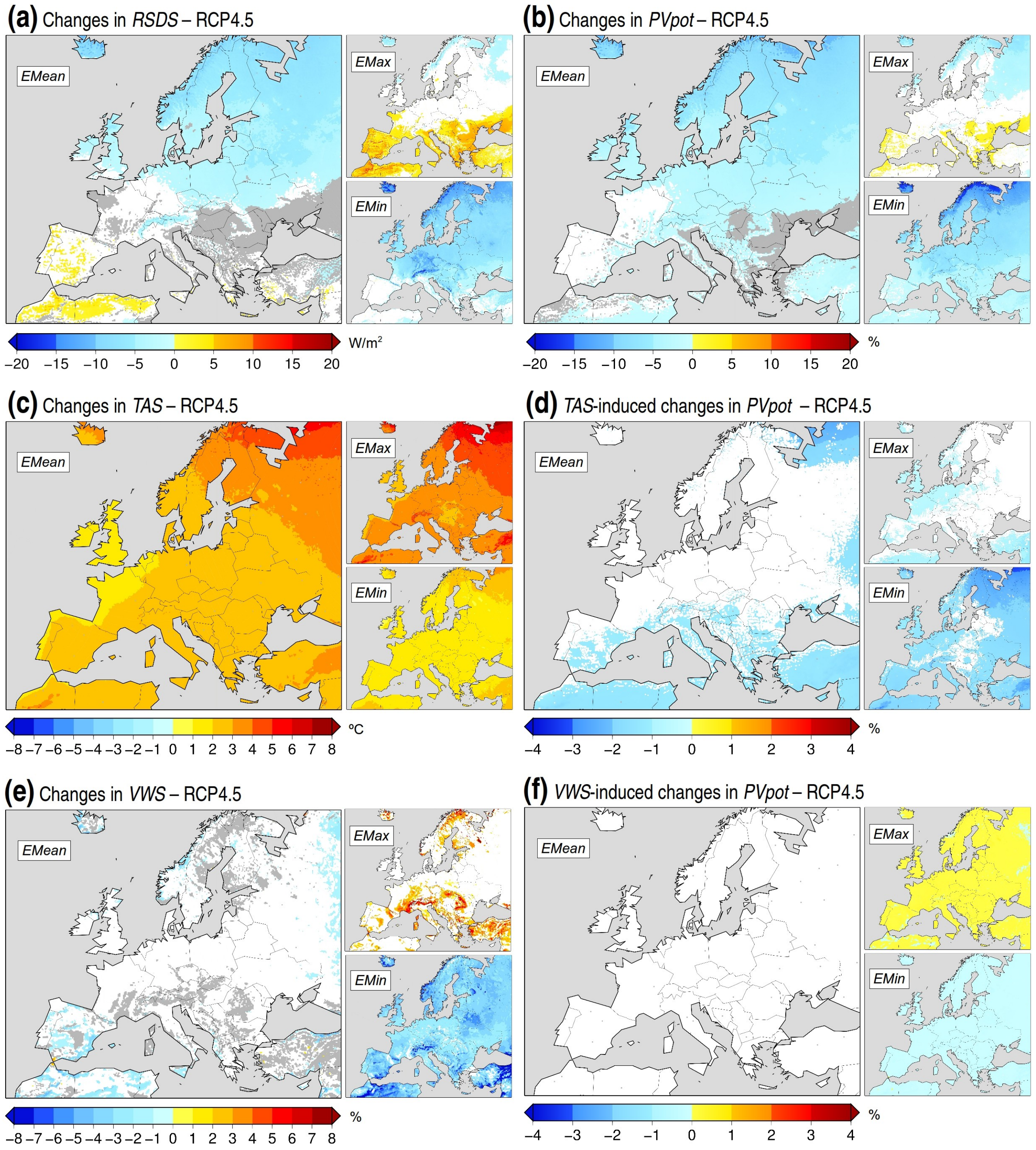

As Fig. 1 for the RCP4.5. Changes projected in the mean values of (a) *RSDS*, (b) *PVpot*, (c) *TAS* and (e) *VWS* under the RCP8.5 to the end of this century (2070-2099 *vs*. 1970-1999) obtained from the *EMean*, *EMax* and *EMin* over land. *EMax* and *EMin* values are colored only if they are significant (p<0.05) within their corresponding ensemble member, otherwise they are depicted in white. *EMean* values are colored only if they are robust, in white if they are negligible and in gray if they are uncertain. (d) and (f) show the *EMean*, *EMax* and *EMin* changes in *PVpot* that would be induced by the changes in either *TAS* alone or *VWS* alone. See Methods for details.

Supplementary Figure 3. Comparison of GCM and RCM projections for *RSDS*.

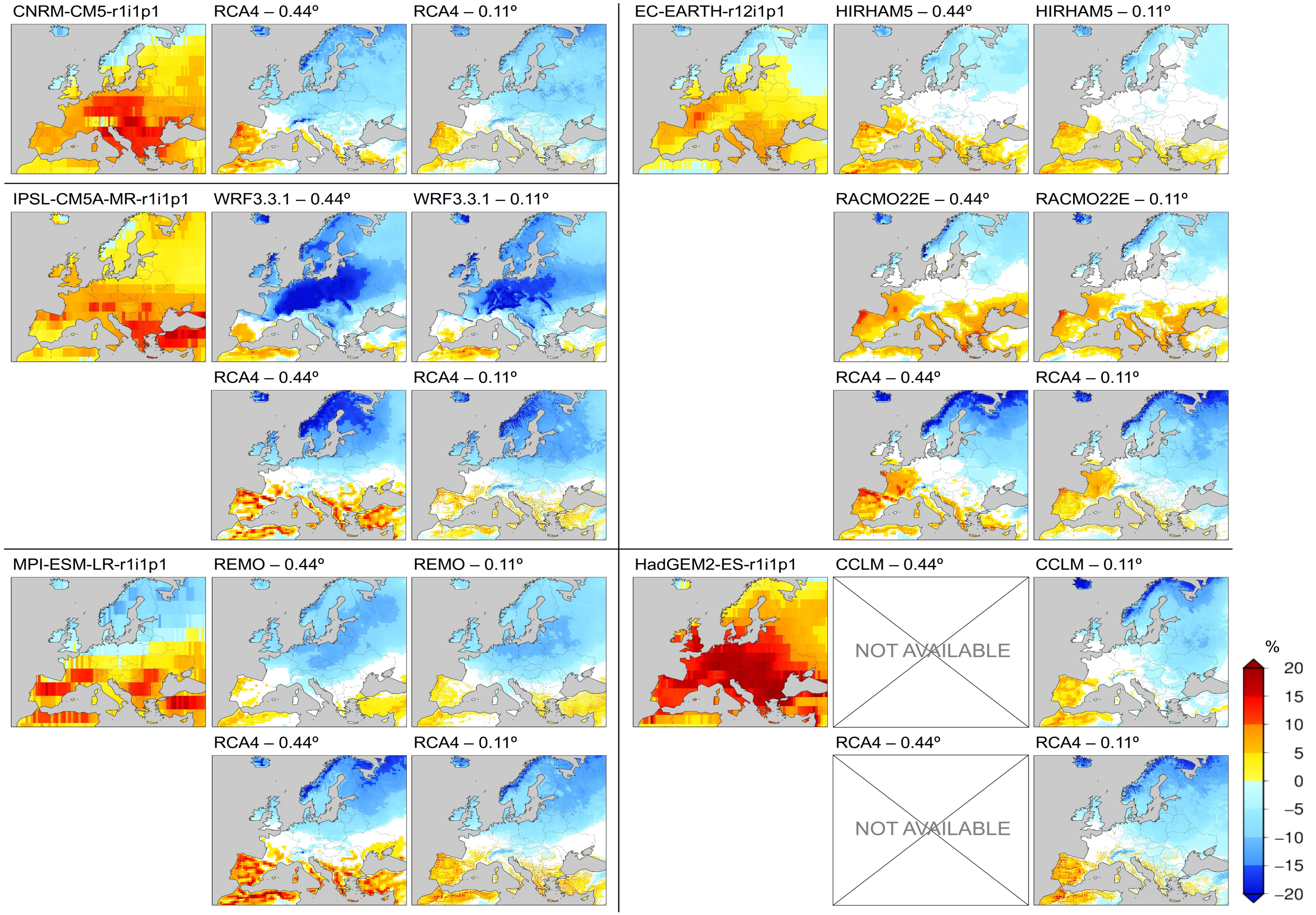

GCM (first and fourth columns) *vs*. GCM RCM-downscaled (two next columns to the right, showing different resolutions of the grid used for the downscaling, 0.44º and 0.11º) *RSDS* projections (2070-2099 minus 1970-1999) under the RCP8.5. Note that the showed EC-EARTH ensemble member, due to availability problems at the date in which this work was performed, is not exactly the same run downscaled by the RCMs.

Supplementary Figure 4. Seasonal climate change projections under RCP8.5.

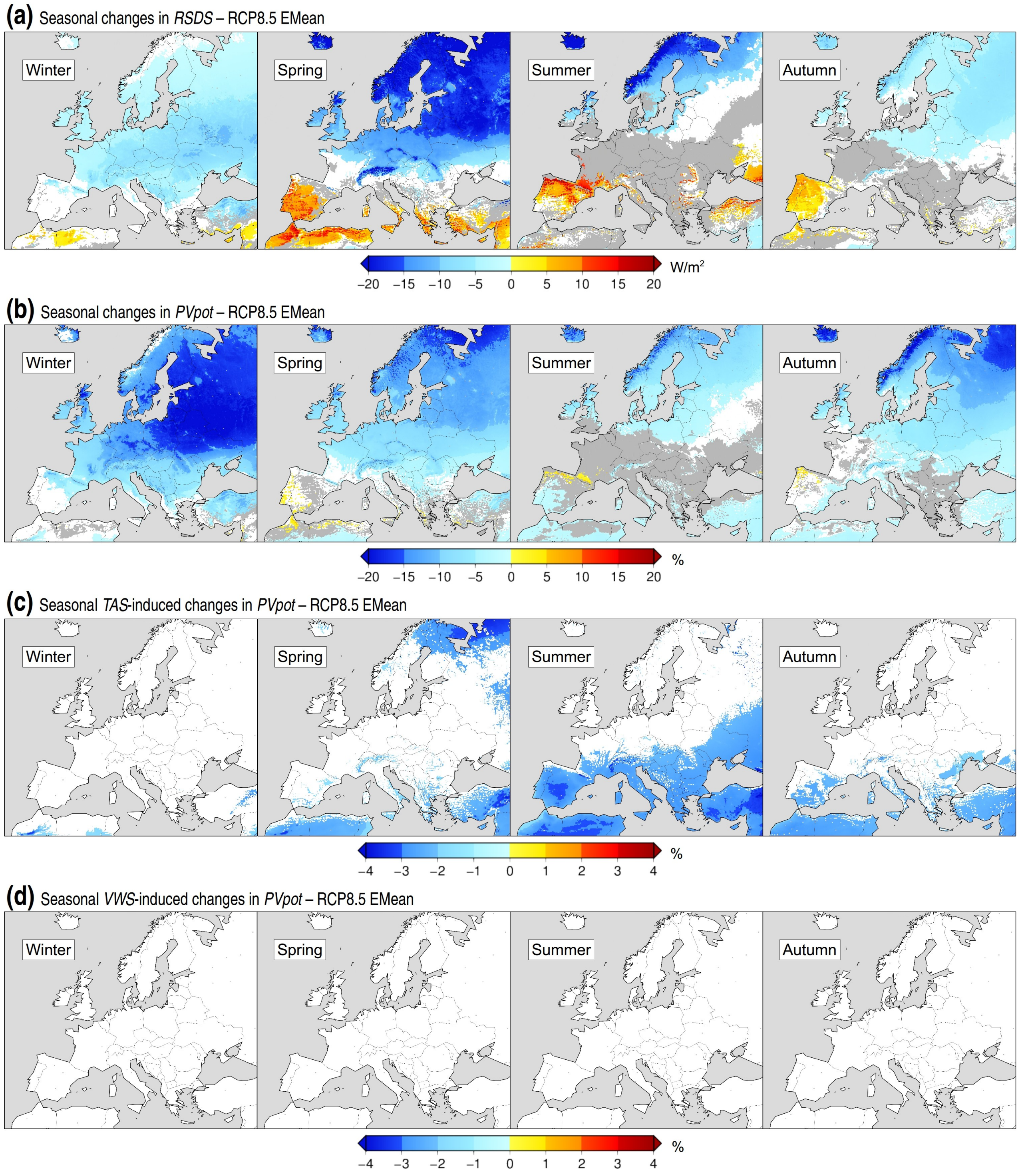

Seasonally, ensemble mean changes projected for the mean values of (a) *RSDS* and (b) *PVpot* under the RCP8.5 to the end of this century (2070-2099 *vs*. 1970-1999) represented following the same approach as in Fig. 1. (c) and (d) show the ensemble mean projected changes in *PVpot* that would be induced by the changes in either *TAS* alone or *VWS* alone by seasons (again, as in Fig. 1). Winter involves December to February, Spring March to May, Summer June to August and Autumn September to November.

Supplementary Figure 5. Seasonal projections for the mean and the time variability of the PV power production series to the end of this century.

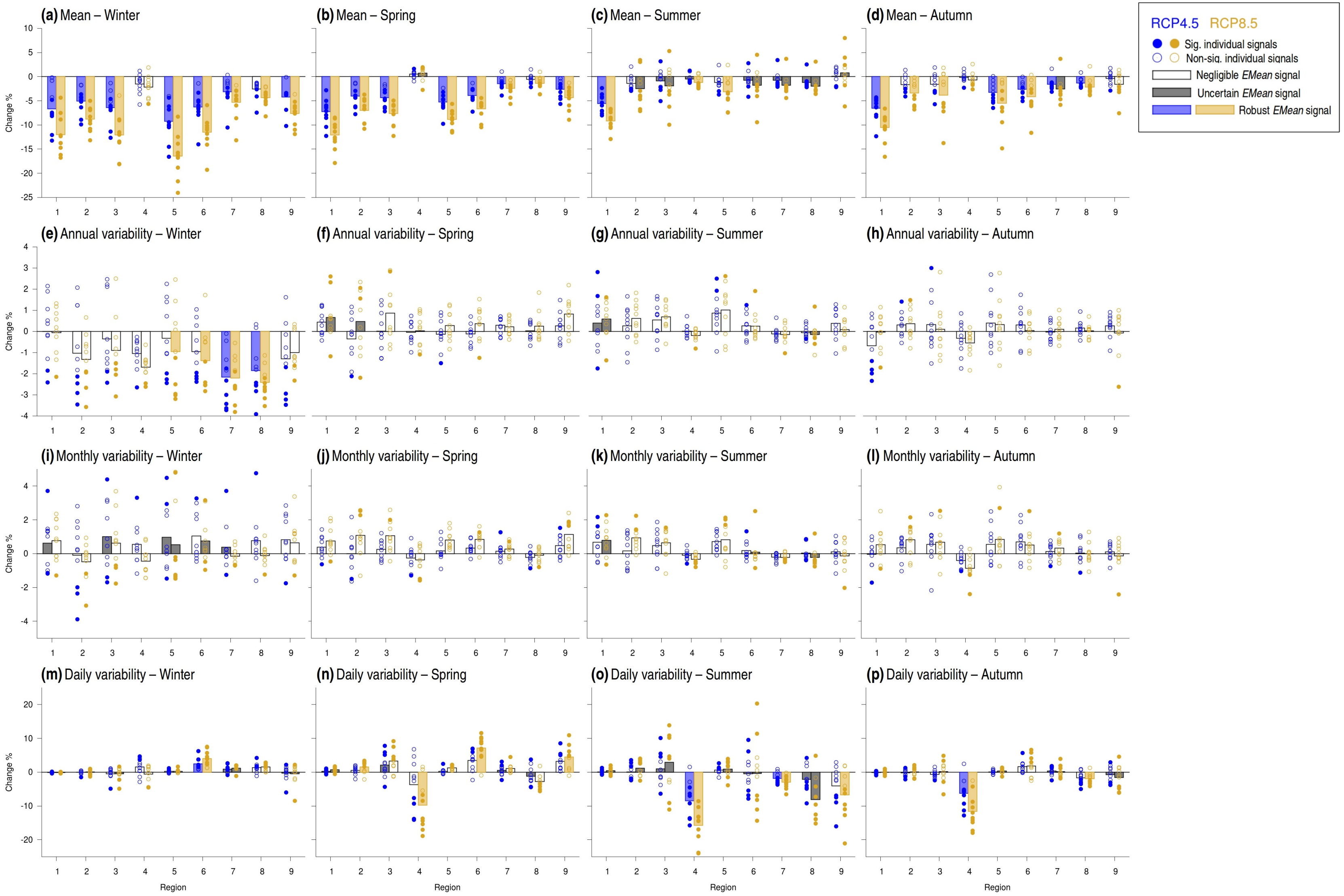

Seasonally, changes projected to the end of this century (2070-2099 *vs.* 1970-1999) in the mean values (1st row) and the annual (2nd row), monthly (3rd row) and daily (4th row) variability of the PV power production series of each region under both the RCP4.5 (blue) and the RCP8.5 (orange). Individual and ensemble mean signals are represented following the same approach as in Fig. 4. Winter involves December to February, Spring March to May, Summer June to August and Autumn September to November.

Supplementary Figure 6. Analysis of the signal-to-noise ratio in the projections for PV power production.

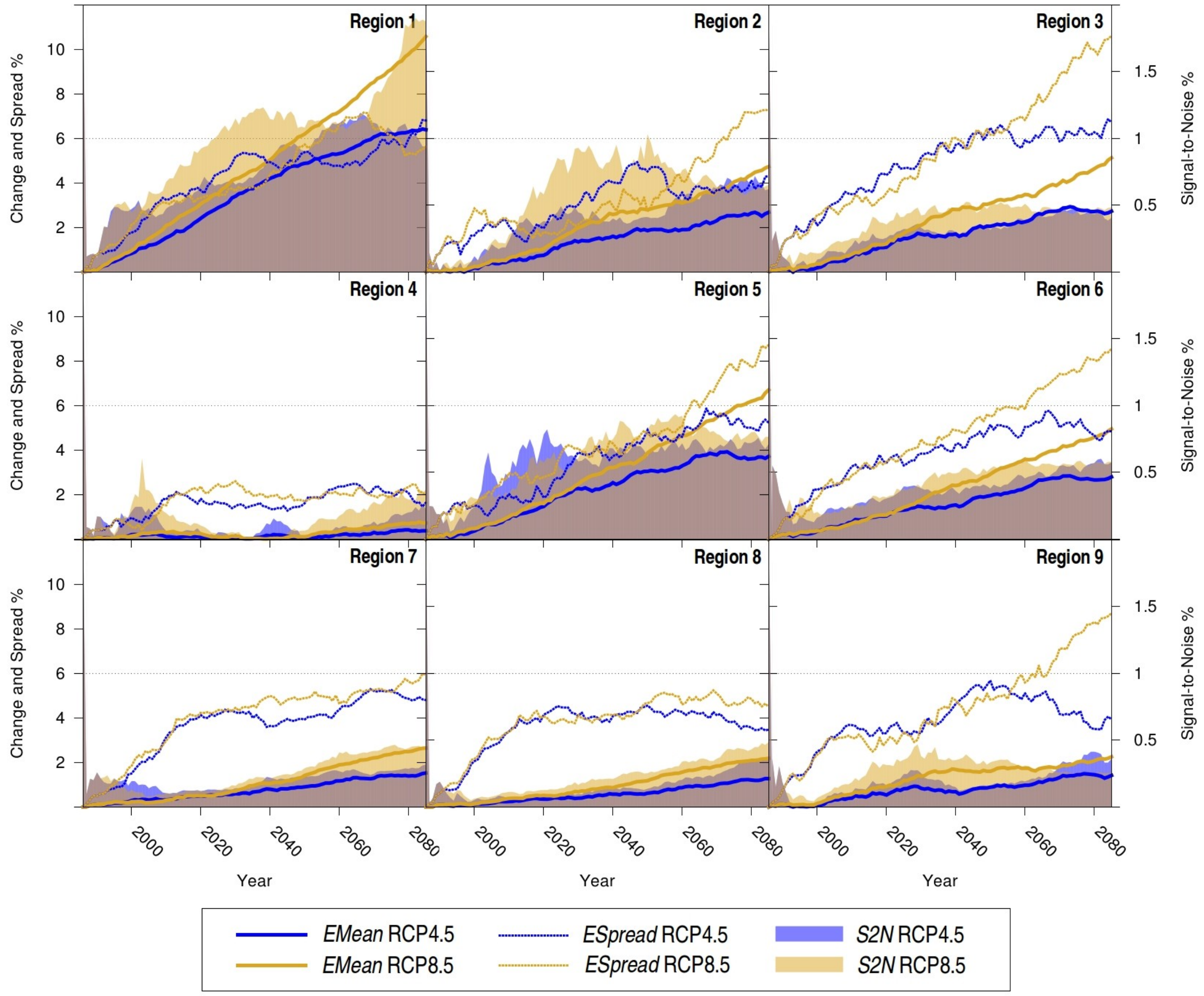

Referred to the left y axes: ensemble mean (solid line) and spread (dashed line) of the 30-year running mean time-series of the estimated PV power production anomalies (expressed in % and in absolute value, *i.e.* what is depicted is the magnitude of the change regardless of its sign) under both the RCP4.5 (blue) and the RCP8.5 (orange) in each region. Referred to the right y axes: the corresponding signal-to-noise ratio (see Methods).

Supplementary Figure 7. Projected changes in time variability.

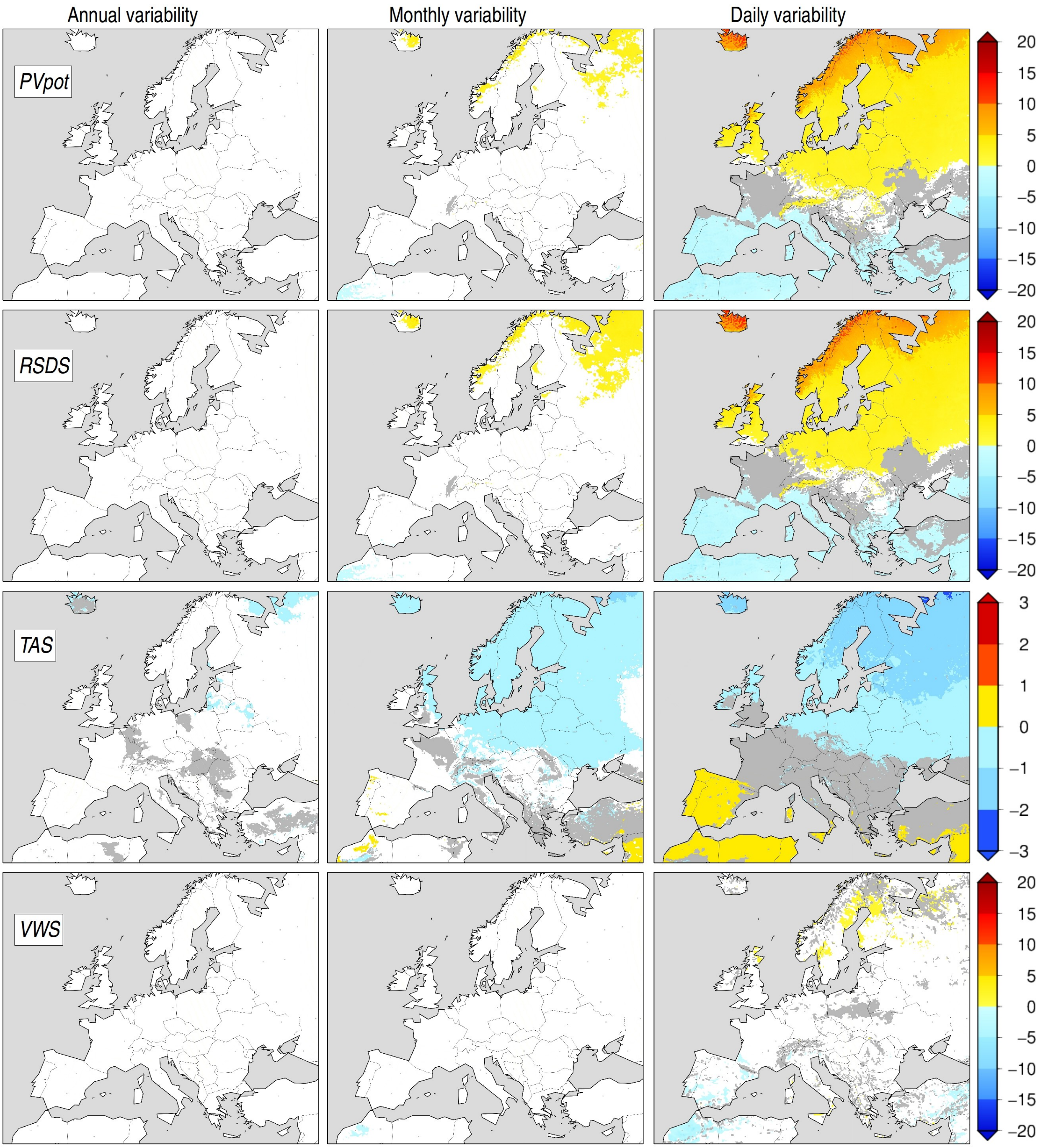

Ensemble mean changes projected for the annual (1st column), monthly (2nd column) and daily (3rd column) variability of the *PVpot* (1st row), *RSDS* (2nd row), *TAS* (3rd row) and *VWS* (4th row) time-series under the RCP8.5 to the end of this century (2070-2099 *vs*. 1970-1999) represented following the same approach as in Fig. 1. Units are % but for *TAS* that is ºC. See Methods for details on the time variability analysis.

Supplementary Figure 8. Projections for the mean and the time variability of the PV power production series by mid-century.

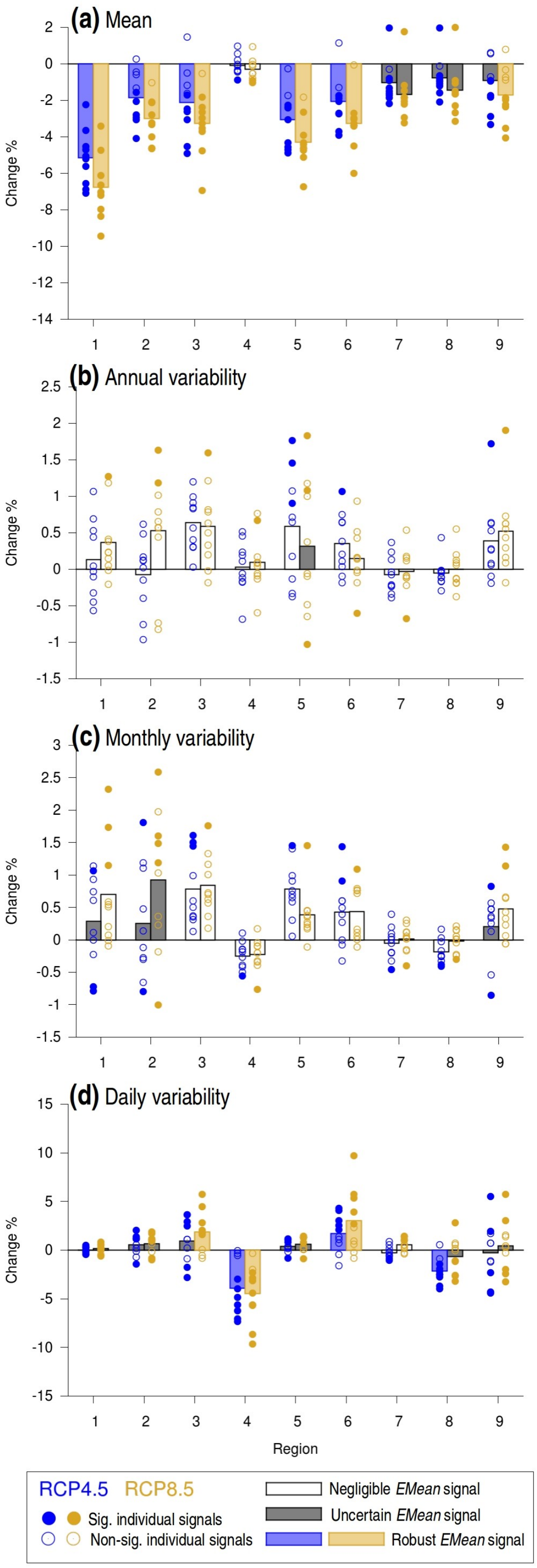

As Fig. 4 but considering the mid-century future period 2040-2069. Changes projected to the mid-century (2040-2069 *vs*. 1970-1999) in (a) the mean, (b) the annual variability, (c) the monthly variability and (d) the daily variability of the PV power production series of each region under both the RCP4.5 (blue) and the RCP8.5 (orange). The individual signals corresponding to each ensemble member are depicted by circles: filled if they are significant, empty if not. Boxes represent ensemble mean signals: colored, white or gray if robust, negligible or uncertain, respectively. See Methods for details.

Supplementary Figure 9. Evaluation of the simulated *RSDS* in the historical period.

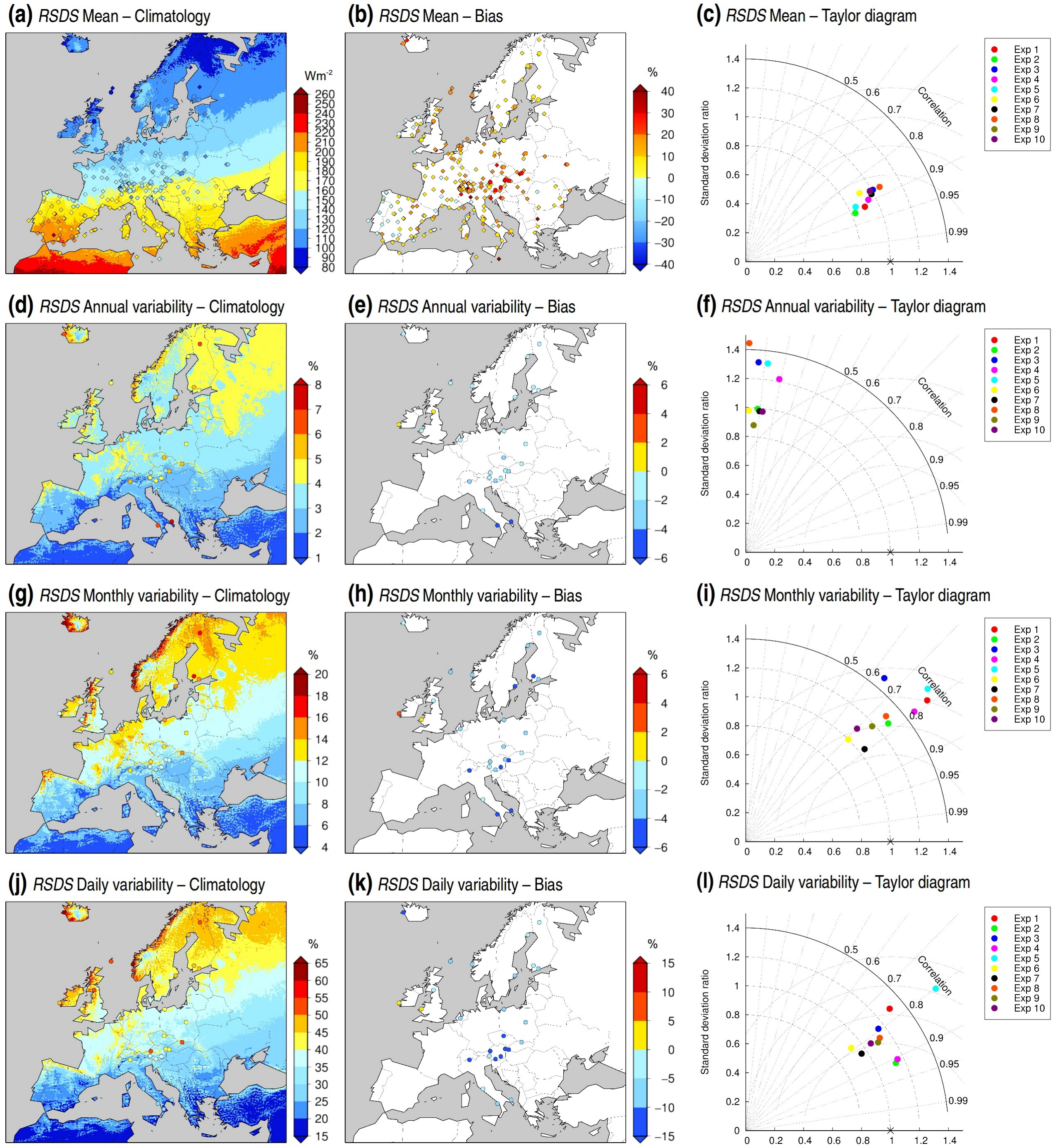

Observed (symbols) and ensemble mean (*EMean*) simulated climatologies in the period 1970-1999 (shadows; left column), *Emean* bias (central column) and Taylor diagrams [36] comparing observed and simulated patterns (right column) of the mean (1st row), the annual variability (2nd column), the monthly variability (3rd row) and the daily variability (4th column) of the *RSDS* time series. See Methods for details on the time variability analysis. Observations were retrieved from the GEBA dataset [37,38] at the monthly time-scale for the period 2000-2005 (represented by diamonds in panels a,b) and from the WRDC dataset [39] at the daily time-scale for the period 1970-1999 (represented by circles). Bias patterns and Taylor diagrams are based on the simulated values during the respective periods.

Supplementary Figure 10. Simulated climatologies of *PVpot*, *TAS* and *VWS* in the historical period.

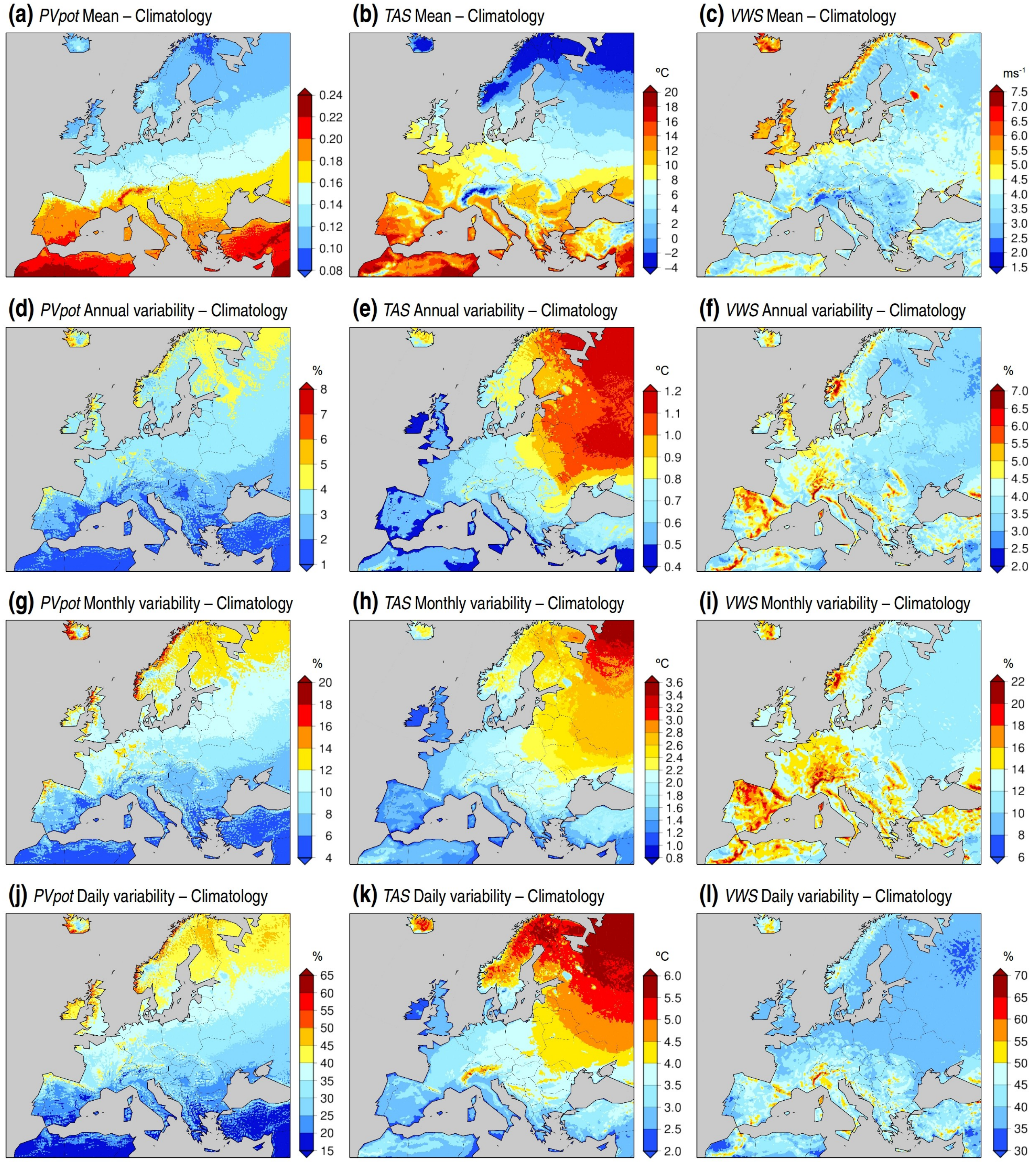

Ensemble mean climatologies in the historical period (1970-1999) of the mean values and the time variability (annual, monthly and daily) of *PVpot*, *TAS* and *VWS*. See Methods for details on the time variability analysis.

Supplementary Figure 11. Estimations of mean PV power production and its time variability assuming the 2050 PV fleet under the current climatology.

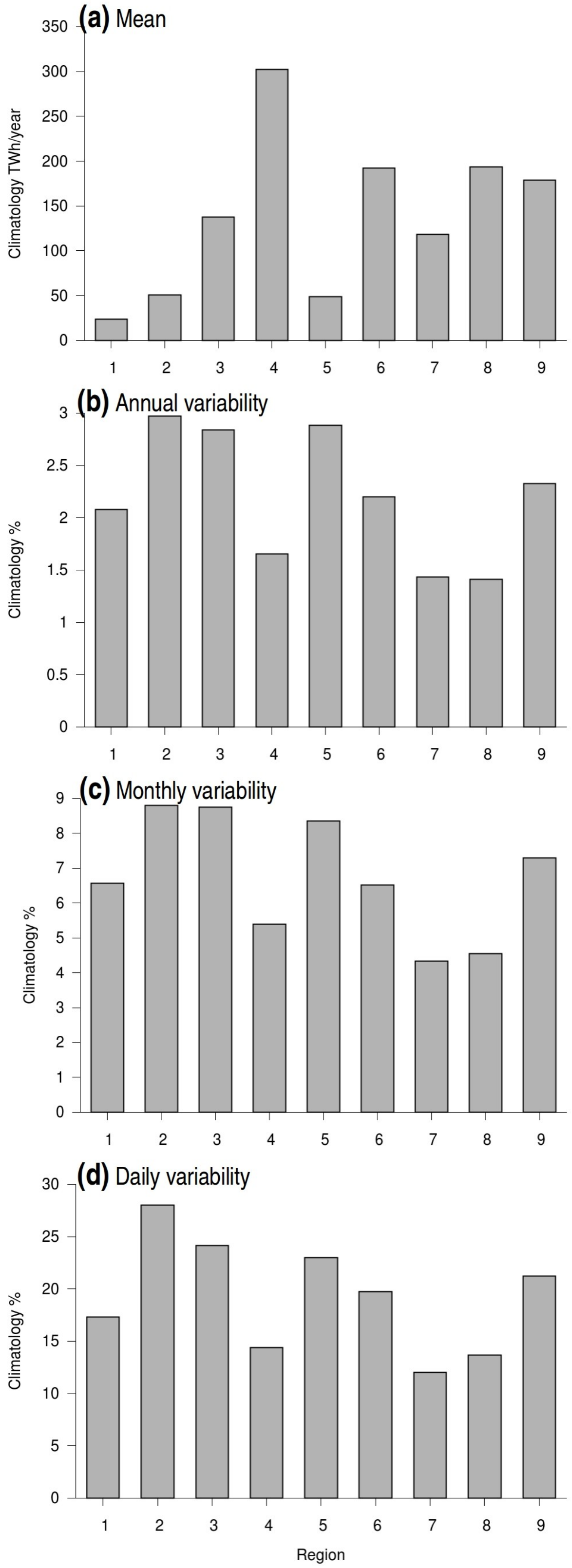

Ensemble mean estimated (a) mean, (b) annual variability, (c) monthly variability and (d) daily variability values of the PV power production series of each region assuming the spatial distribution of PV units presented in Fig. 2 and the simulated climatologies for the historical period (1970-1999).

Supplementary Table 1. Overview of the analyzed EURO-CORDEX experiments.

Exp. ID	Forcing GCM run	RCM	Institution (abbreviation)	Land-surface scheme	Planetary boundary layer scheme	Convection scheme	Microphysics scheme	Radiation scheme	No. of vertical levels
1	ICHEC-EC-EARTH-r3i1p1	HIRHAM 5	Danish Meteorological Institute (DMI)	[1]	[8]	[13]	[20]	[28] [29]	31
2	MOHC-HadGEM2-ES-01	CCLM 4.8.17	CLM Community (CLMCOM)	TERRA-ML [2]	[8]	[13]	[2] [21]	[30]	40
3	IPSL-CM5A-MR-r1i1p1	WRF 3.3.1	Institute Pierre Simon Laplace / Institut National de l'Environment Industriel et des Risques (IPSL-INERIS)	NOAH [3]	YSU [9]	[14]	[22]	RRTMG [31]	32
4	ICHEC-EC-EARTH-r1i1p1	RACMO 2.2	Royal Netherlands Meteorological Institute (KNMI)	[4] [5]	[10] [11]	[13] [15] [16]	[23] [24] [25] [26]	[32] [33]	40
5	MPI-ESM-LR-r1i1p1	REMO 2009	Climate Service Center (CSC)	[1] [6]	[8]	[13] [15] [17]	[20]	[28] [29]	27
6	CNRM-CM5-r1i1p1	RCA 4	Swedish Meteorological and Hydrological Institute (SMHI)	[7]	[12]	[18] [19]	[27]	[34] [35]	40
7	ICHEC-EC-EARTH-r12i1p1	RCA 4	Swedish Meteorological and Hydrological Institute (SMHI)	[7]	[12]	[18] [19]	[27]	[34] [35]	40
8	IPSL-CM5A-MR-r1i1p1	RCA 4	Swedish Meteorological and Hydrological Institute (SMHI)	[7]	[12]	[18] [19]	[27]	[34] [35]	40
9	MOHC-HadGEM2-ES-01	RCA 4	Swedish Meteorological and Hydrological Institute (SMHI)	[7]	[12]	[18] [19]	[27]	[34] [35]	40
10	MPI-ESM-LR-r1i1p1	RCA 4	Swedish Meteorological and Hydrological Institute (SMHI)	[7]	[12]	[18] [19]	[27]	[34] [35]	40

From left to right: experiment identifier (ID), GCM driving run, RCM, provider institution and RCM experimental setup details. Each experiment comprises one historical and two scenario (RCP4.5 and RCP8.5) runs, spanning the periods 1970-2005 and 2006-2099 respectively. The horizontal resolution of all simulations is 0.11° in both latitude and longitude.

Supplementary References

1. Hagemann, S. An improved land surface parameter dataset for global and regional climate models. MPI for Meteorology, Hamburg, Report No. 336 (2002).

2. Doms, G. *et al.* A description of the nonhydrostatic regional model LM. Part II: physical parameterization. Available at: http://www.cosmomodel.org/content/ model/documentation/core/cosmoPhysParamtr.pdf (2007).

3. Ek, M. B. *et al.* Implementation of Noah land surface model advances in the National Centers for Environmental Prediction operational mesoscale Eta model. *J. Geophys. Res.* **108,** 8851 (2003).

4. van den Hurk, B. J. J. M., Viterbo, P., Beljaars, A. C. M., Betts, A. K. Offline validation of the ERA40 surface scheme. ECMWF Tech. Report No. 75 (2000).

5. Balsamo, G. P. *et al.* A revised hydrology for the ECMWF model: Verification from field site to terrestrial water storage and impact in the Integrated Forecast System. *J. Hydrometeor.* **10,** 623–643 (2009).

6. Rechid, D., Raddatz, T., Jacob, D. Parameterization of snow- free land surface albedo as a function of vegetation phenology based on MODIS data and applied in climate modelling. *Theor. Appl. Climatol.* **95,** 245–255 (2009).

7. Samuelsson, P., Gollvik, S., Ullerstig, A. The land-surface scheme of the Rossby Centre regional atmospheric climate model (RCA3). *SMHI Rep. Met.* **122,** 25 (2006).

8. Louis, J.-F. A parametric model of vertical eddy fluxes in the atmosphere. *Bound. Layer Meteorol.* **17,** 187–202 (1979).

9. Hong, S.-Y., Noh, Y., Dudhia, J. A new vertical diffusion package with an explicit treatment of entrainment processes. *Mon. Weather. Rev.* **134,** 2318–2341 (2006).

10. Lenderink, G., Holtslag, A. A. M. An updated length-scale formulation for turbulent mixing in clear and cloudy boundary layers. *Q. J. R. Meteorol. Soc.* **130,** 3405–3427 (2004).

11. Siebesma, A.P., Soares, P. M. M., Teixeira, J. A Combined Eddy-Diffusivity Mass-Flux Approach for the Convective Boundary Layer. *J. Atmos. Sci.* **64,** 1230–1248 (2007).

12. Cuxart, J., Bougeault, P., Redelsperger, J.-L. A turbulence scheme allowing for mesoscale and large-eddy simulations. *Q. J. R. Meteorol. Soc.* **126,** 1–30 (2000).

13. Tiedtke, M. A comprehensive mass flux scheme for cumulus parameterization in large-scale models. *Mon. Weather Rev.* **117,** 1779–1799 (1989).

14. Grell, G. A., Devenyi, D. A generalized approach to parameterizing convection combining ensemble and data assimilation techniques. *Geophys. Res. Lett.* **29,** 38-1–38-4 (2002).

15. Nordeng, T. E. Extended versions of the convection parametrization scheme at ECMWF and their impact upon the mean climate and transient activity of the model in the tropics. ECMWF Tech. Memo. No. 206 (1994).

16. Neggers, R. A. J., Koehler, M., Beljaars, A. C. M. A dual mass flux framework for boundary layer convection, Part I: Transport. *J. Atmos. Sci.* **66,** 1465–1487 (2009).

17. Pfeifer, S. Modeling cold cloud processes with the regional climate model REMO. MPI for Meteorology, Hamburg, Reports on Earth System Science No. 23 (2006).

18. Kain, J. S., Fritsch, J. M. A one-dimensional entraining/detraining plume model and its application in convective parameterization. *J. Atmos. Sci.* **47,** 2784–2802 (1990).

19. Kain, J. S., Fritsch, J. M. Convective parameterization for mesoscale models: the Kain-Fritsch scheme. The representation of cumulus convection in numerical models. *Meteorol. Monogr.* **24,** 165–170 (1993).

20. Lohmann, U., Roeckner, E. Design and performance of a new cloud microphysics scheme developed for the ECHAM general circulation model. *Clim. Dyn.* **12,** 557–572 (1996).

21. Baldauf, M., Schulz, J. P. Prognostic precipitation in the Lokal- Modell (LM) of DWD. *COSMO Newslett.* **4,** 177–180 (2004).

22. Hong, S.-Y., Dudhia, J., Chen, S.-H. A revised approach to microphysical processes for the bulk

parameterization of cloud and precipitation. *Mon. Weather Rev.* **132,** 103–120 (2004).

23. Tiedtke, M. Representation of clouds in large-scale models. *Mon. Weather. Rev.* **121,** 3040–3061 (1993).

24. Tompkins, A. M., Gierens, K., Rädel, G. Ice supersaturation in the ECMWF Integrated Forecast System. *Q. J. R. Meteorol. Soc.* **133,** 53–63 (2007).

25. ECWMF-IFS: IFS documentation-Cy31r1. PART IV: Physical Processes. Available at: http://www.ecmwf.int/research/ifsdocs/ CY31r1/PHYSICS/IFSPart4.pdf (2007).

26. Neggers, R. A. J. A dual mass flux framework for boundary layer convection. Part II: Clouds. *J. Atmos. Sci.* **66,** 1489–1506 (2009).

27. Rasch, P. J., Kristjánsson, J. E. A comparison of the CCM3 model climate using diagnosed and predicted condensate parameterizations. *J. Climate* **11,** 1587–1614 (1998).

28. Morcrette, J. J., Smith, L., Fouquart, Y. Pressure and temperature dependence of the absorption in longwave radiation parameterizations. *Beitr. Phys. Atmos.* **59,** 455–469 (1986).

29. Giorgetta, M., Wild, M. The water vapor continuum and its representation in ECHAM4. MPI for Meteorology, Hamburg, Report No. 162 (1995).

30. Ritter, B., Geleyn, J.-F. A comprehensive radiation scheme of numerical weather prediction with potential application to climate simulations. *Mon. Weather Rev.* **120,** 303–325 (1992).

31. Iacono, M. J. *et al.* Radiative forcing by long-lived greenhouse gases: calculations with the AER radiative transfer models. *J. Geophys. Res.* **113,** D13103 (2008).

32. Fouquart, Y., Bonnel, B. Computations of solar heating of the earth's atmosphere: A new parameterization. *Beitr. Phys. Atmos.* **53,** 35–62 (1980).

33. Mlawer, E. J., Taubman, S. J., Brown, P. D., Iacono, M. J., Clough, S. A. Radiative transfer for inhomogeneous atmospheres: RRTM, a validated correlated-k model for the longwave. *J. Geophys. Res.* **102D,** 16663–16682 (1997).

34. Savijärvi, H. A fast radiation scheme for mesoscale model and short-range forecast models. *J. Appl. Meteorol.* **29,** 437–447 (1990).

35. Sass, B. H., Rontu, L., Savijaärvi, H., Räisänen, P. HIRLAM- 2 radiation scheme: documentation and tests. SMHI HIRLAM Technical Report No. 16 (1994).

36. Taylor, K. E. Summarizing multiple aspects of model performance in a single diagram. *J. Geophys. Res.* **106(D7),** 7183-7192 (2001).

37. Gilgen, H., Ohmura, A. The global energy balance archive. *B. Am. Meteorol. Soc.* **80(5),** 831-850 (1999).

38. Wild, M., Folini, D., Schär, C., Loeb, N., Dutton, E. G., König-Langlo, G. The global energy balance from a surface perspective. *Clim. Dyn.* **40**, 3107-3134 (2013).

39. World Radiation Data Centre, http://wrdc.mgo.rssi.ru/
